# Supplementary material for: Heat current-driven topological spin texture transformations and helical q-vector switching
Source: Nat Commun. 2023 Nov 4;14:7094. doi: 10.1038/s41467-023-42846-7 (PMC10625536; doi:10.1038/s41467-023-42846-7)
Supplement: Supplementary file 1 — Supplementary Information [file 41467_2023_42846_MOESM1_ESM.pdf]

Supplemental information for "Heat current-driven topological spin texture transformations and helical q-vector switching."

Fehmi Sami Yasin*[1], Jan Masell[1,2], Kosuke Karube[1], Daisuke Shindo[1], Yasujiro Taguchi[1], Yoshinori Tokura[1,3,4], and Xiuzhen Yu*[1]

[1] RIKEN Center for Emergent Matter Science (CEMS), Wako, Japan.

[2] Institute of Theoretical Solid State Physics, Karlsruhe Institute of Technology (KIT), Karlsruhe, Germany

[3] Department of Applied Physics, University of Tokyo, Tokyo, Japan.

[4] Tokyo College, University of Tokyo, Tokyo, Japan

* Email: fehmi.yasin@riken.jp; yu_x@riken.jp

Supplemental Figures

Figure S1 shows a selection of temperature gradient devices fabricated using a focused ion beam apparatus (Thermofisher Helios 5 Dual Beam and Hitachi NB5000). Fig. S1a shows a schematic illustrating the main features of the device shown in the scanning electron microscope (SEM) micrograph in Fig. S1b. The left-hand-side (hot) is elevated above the silicon chip base (with a thin SiO_2 surface layer) and is composed of glass (SiO_2, colored gray in Fig. S1a). Using Ga ion beam Pt deposition, we deposited a Pt wire heater (light gray) in a winding pattern to increase the resistance of the wire, which is attached to Pt electrodes located at the front and back ends of the glass base. We attached the $(Fe_{0.63}Ni_{0.3}Pd_{0.07})_3P$ (FNPP, blue) thin plate to this base using Pt deposition and attached the right-hand side (cold) of the device directly to the Si chip base (with a thin SiO_2 surface layer) which acts as a cold bath, after depositing a layer of insulating $SiC_8H_{20}O_4$ (TEOS) composition (orange) to prevent any electrical connection to the cold bath. The FNPP thin plate was positioned over a TEM viewing window and was thinned to the final thickness ($t \approx 150$ nm) after the final ion beam Pt/TEOS depositions were performed to avoid any redeposition on the thin plate's surface. Figure S1c-f show SEM micrographs of two similar devices and their side-view profiles (Fig. S1d, f). These devices had (Fig. S1c-d) Si and (Fig. S1e-f) C bases, respectively, instead of glass as well as W wire heaters instead of Pt. The results shown in the main text were reproduced in all three devices.

Figure S2a shows the magnetic state diagram measured within a sample of the same geometry (shown in the SEM micrograph in Fig. S2b) as the devices used for the temperature gradient experiments. The magnetic state diagram was generated by heating to the target temperature at zero field and then sweeping the field from zero to the maximum field shown in the plot, taking Lorentz transmission electron microscopy (LTEM) micrographs at each temperature and field value to determine the spin textures present. The overlayed colour plotting represents the non-helical spin texture density, including skyrmions (Sky), antiskyrmions (Asky) and non-topological bubbles (NTB).

Figure S3 shows four LTEM micrograph still frames captured as ∇T was being increased across the sample, resulting in the motion of the magnetic fork defect marked by a green

arrow. The color bar at the bottom of the figure represents relative temperature, with blue indicating cold (left-hand-side) and red indicating hot (right-hand-side).

Figure S4 shows a selection of LTEM micrographs while uniformly heating the thin plate pictured in Extended Data Fig. 1b from $T_0 = 293$ K to $T = 393$ K before cooling it back to $T_0 = 293$ K at heating/cooling rates of $\frac{dT}{dt} = 5\ \mathrm{K\,s^{-1}}$, $10\ \mathrm{K\,s^{-1}}$, and $50\ \mathrm{K\,s^{-1}}$. The initial double-**q** vector magnetic helical state reemerges upon cooling the sample for all three heating/cooling rates. This implies that the alignment of the double-**q** vector state into a single-**q** vector state must be driven by a $\boldsymbol{\nabla}T$-related mechanism, such as the one we hypothesize in the current work.

Figure S5 shows the unidirectional $\boldsymbol{\nabla}T$-driven transformation from Askys to NTBs to Skys while under an externally applied magnetic field with magnitude $|B_z| = 439$ mT. The initial magnetic state, shown in the LTEM micrograph in Fig. S5a, consists of Askys and NTBs. These spin textures transform into NTBs and Skys as $\boldsymbol{\nabla}T$ increases to (b) $\boldsymbol{\nabla}T = 9.4 \times 10^{-2}\ \mathrm{K\,\mu m^{-1}}$ and (c) $\boldsymbol{\nabla}T = 3.3 \times 10^{-1}\ \mathrm{K\,\mu m^{-1}}$, respectively. This can be explained in terms of the relative total energy of Askys and Skys under application of an external field. The numerically calculated energy relative to the ferromagnetic state (FM) of Askys and Skys ($E - E_{FM}$) is plotted in Fig. S5d versus magnetization M_s. Note that increasing M_s corresponds to decreasing T. These calculations reveal that Skys have a lower energy compared to Askys for all temperatures while an external magnetic field is applied. By applying $\boldsymbol{\nabla}T$, the thermal energy added to the Asky state is enough to perturb the spin texture and initiate a transformation towards the lower energy Sky state.

Figure S6 and Fig. S7 estimate the $\boldsymbol{\nabla}T$ across the thin plate devices used in this study. We applied a current I_{heater} to the heater wire until the magnetic contrast in LTEM began to disappear at the hot end (nearest to the wire) of the thin plate, which is the upper right of the LTEM micrographs in Fig. S6a-h and the left-hand-side of Fig. S7a-i. As shown in these micrographs, the loss of contrast occurred along a clear line (drawn by the dashed orange and red line in Fig. S6 and S7, respectively) perpendicular to the temperature gradient direction and was the result of the magnet heating above T_C and thus entering the paramagnetic regime. As expected, this T_C line propagates further towards the cold end of the sample as I_{heater} increases and we used it to measure the location of T_C as a function of I_{heater}. Using these measured distances, we calculated $\boldsymbol{\nabla}T(I_{heater})$ assuming that $T_{cold\ bath} = 295$ K and plot the resulting data along with an I_{heater}^2 fit in Fig. S6i and Fig. S7j, respectively.

Note on helical q-vector orientation

The magnetic helices intrinsic to FNPP have modulation **q**-vectors aligned at a finite angle to the [110] and [$\bar{1}$10] crystal axes. This contrasts with the helical **q**-vectors in Heusler magnets with D_{2d} crystal symmetry, which are pinned to the [100] and [010] crystal axes. The specific pinning to these axes in D_{2d} stems from the additional rotational symmetries with respect to the [100] and [010] crystal axes. This manifests as an additional parameter in the DMI energy for S_4 systems whereas D_{2d} systems only have a single DMI parameter: [1]

$$\begin{aligned} H_{S_4} = &-D_1\left(n_y\partial_x n_z - n_z\partial_x n_y\right) + D_1\left(n_z\partial_y n_x - n_x\partial_y n_z\right) \\ &-D_2(n_z\partial_x n_x - n_x\partial_x n_z) + D_2\left(n_z\partial_y n_y - n_y\partial_y n_z\right) \end{aligned} \qquad 1$$

and

$$H_{D_{2d}} = -D\left(n_y \partial_x n_z - n_z \partial_x n_y\right) + D\left(n_z \partial_y n_x - n_x \partial_y n_z\right), \quad 2$$

where D, D_1, and D_2 are the DMI coefficients, and n_i is the ith component of the normalized magnetization. In the D_1 terms of H_{S_4} and both $H_{D_{2d}}$ terms, the energy is minimized for Bloch twists of the magnetization along x (righthanded) and y (lefthanded), i.e., there is an energy penalty for twists that deviate from the yz-plane along the $\hat{x}$-direction or from the xz-plane along the $\hat{y}$-direction. The D_2 terms in H_{S_4}, however, favour Néel twists, i.e., twists within the xz-plane along the $\hat{x}$-direction or in the yz-plane along the $\hat{y}$-direction, clockwise along x and counterclockwise along y. If both D_1 and D_2 are nonzero, the DMI energy is minimized with Bloch twists along a **q**-vector oriented at an angle away from x and y. As this angle can be clearly observed in Fig. 2a in the main text, we conclude that both D_1 and D_2 are nonzero in FNPP, where mostly D_2 is at work as it pins the helices in the $[110]$ and $[\bar{1}10]$ directions. Note, however, that in isotropic systems both energy functionals are equivalent up to a rotation of the coordinate system which we exploited for our simulations.

1. Bogdanov, A. N. & Yablonskii, D. A. Thermodynamically stable 'vortices' in magnetically ordered crystals. The mixed state of magnets. *Zh. Eksp. Teor. Fiz* **95**, 178 (1989).

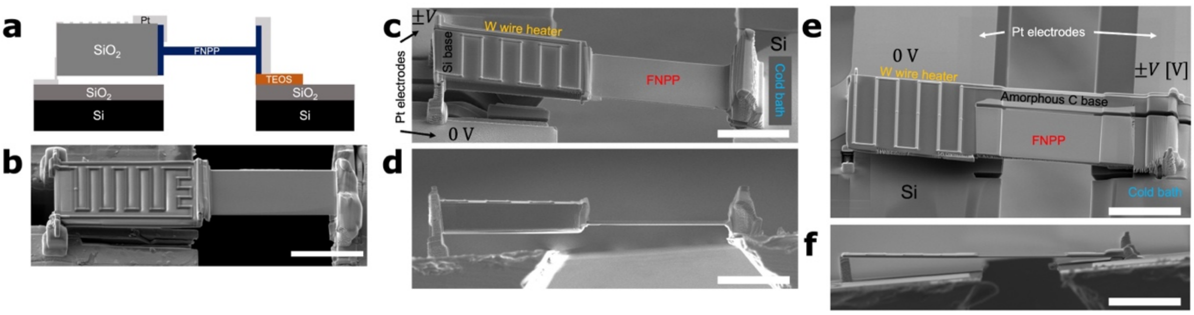

Figure S1: Heat-current devices composed of $Fe_{1.9}Ni_{0.9}Pd_{0.2}P$ (FNPP). a, Schematic of FIB-fabricated device enabling a temperature gradient across the FNPP viewing window. A Pt wire heater was deposited via ion beam-gas deposition onto an insulating SiO_2 base (left-hand-side) that is connected to the FNPP thin plate via Pt-deposition on one side and elevated above a Si chip (with a thin SiO_2 surface layer) below. The FNPP thin plate is connected to the Si chip cold bath on the right-hand side (separated by an insulating TEOS layer to ensure electrical isolation of the thin plate) which is held at room temperature. **b**, SEM micrograph of the device, clearly showing the Pt pillar electrodes which are connected to the wire heater. **c-f**, SEM micrographs of two similar devices and their side-view profiles (d, f). These devices had (c-d) Si and (e-f) C bases, respectively, instead of glass as well as W wire heaters instead of Pt. Scalebars in (b-f) are 10 μm.

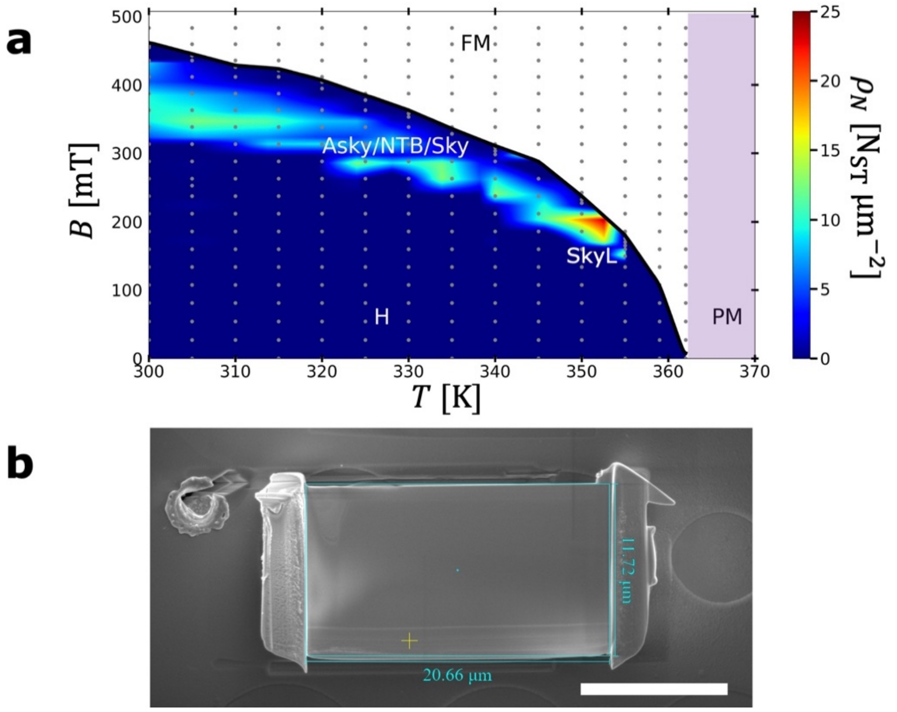

Figure S2: Magnetic state diagram in a $Fe_{1.9}Ni_{0.9}Pd_{0.2}P$ thin plate. **a**, Density of spin textures as a function of temperature T [K] and applied magnetic field B [mT]. The spin textures in the measurement include antiskyrmions (Asky), nontopological bubbles (NTB), and skyrmions (Sky). Spin texture density is represented by the colormap shown on the right-hand-side, with dark blue representing $\rho_N = 0$ (lowest energy helical magnetic state) and dark red representing the maximum ρ_N observed in the thin plate, corresponding to the skyrmion lattice state (SkyL). The purple area shows the paramagnetic state (PM) at $T > T_C \approx 362$ K, while the white area shows the ferromagnetic state (FM) in which the spins are polarized along the same direction as the applied magnetic field when applied above the saturation field illustrated by the boundary drawn by the solid black line. The circular markers represent the temperature and field values at which real space LTEM images were acquired to measure the spin texture density and magnetic states present. **b**, SEM micrograph showing a top-down view of the thin plate which is ≈ 12 µm × 21 µm in area and has a thickness of ≈ 150 nm, which is approximately the same size as the thin plates in the temperature gradient devices. Scale bar 10 µm.

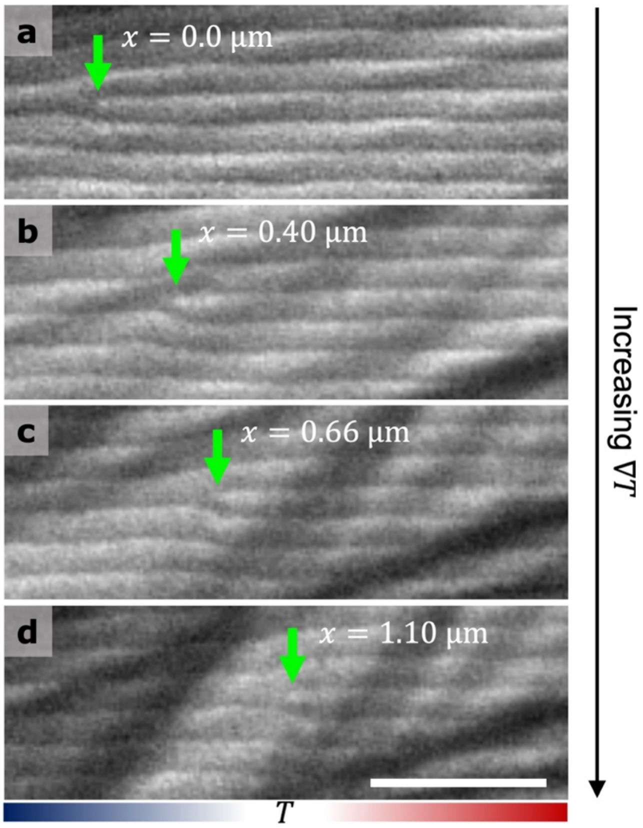

Figure S3. $\boldsymbol{\nabla T}$-driven magnetic defect motion from cold to hot. Magnetic dislocation defect embedded within the helices propagates from the cold to the hot region of the sample with increasing $\boldsymbol{\nabla} T$. The green colored arrow tracks the magnetic defect as it moves from (**a**) $x = 0\ \mu m$ to (**b**) $x = 0.42\ \mu m$ to (**c**) $x = 0.96\ \mu m$ to (**d**) $x = 1.33\ \mu m$. The scale bar is $1\ \mu m$.

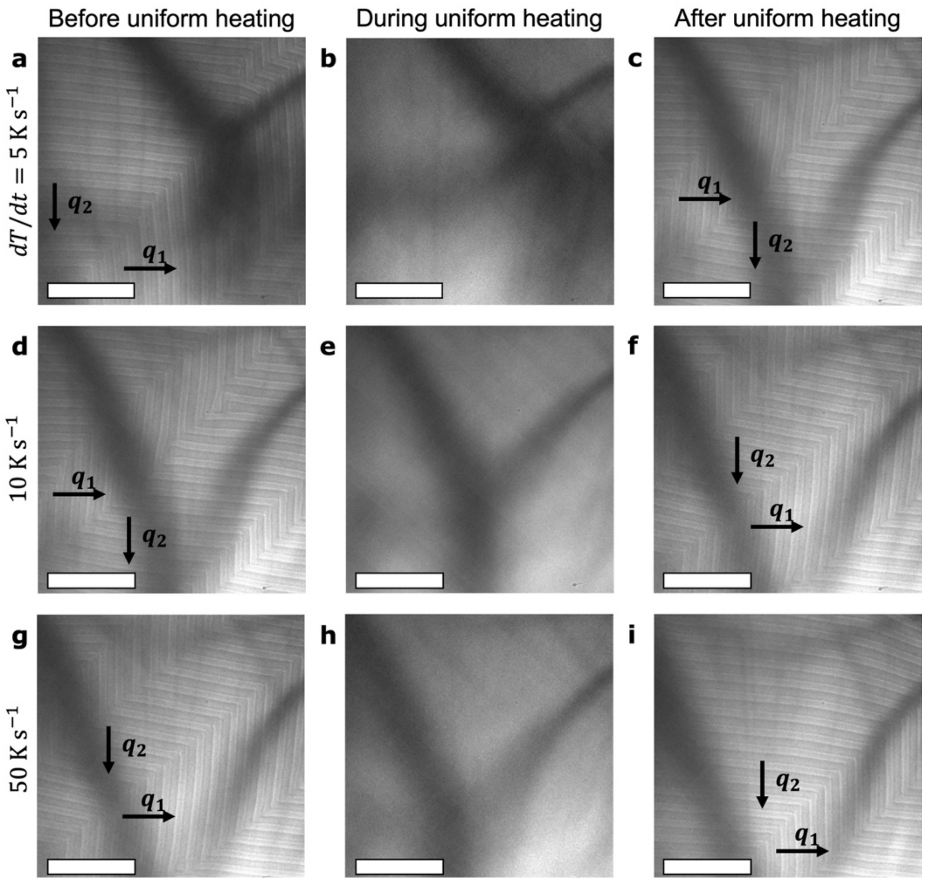

Figure S4: Uniform heating of magnetic ground state in a $Fe_{1.9}Ni_{0.9}Pd_{0.2}P$ thin plate. **a-i**, LTEM micrographs (a, d, g) before, (b, e, h) during, and (c, f, i) after uniform heating from $T_0 = 293$ K to $T_f = 393$ K, and then cooling back to $T_0 = 293$ K at a rate of (a-c) $dT/dt = 5\ \mathrm{K\,s^{-1}}$, (d-f) $dT/dt = 10\ \mathrm{K\,s^{-1}}$, and (g-i) $dT/dt = 50\ \mathrm{K\,s^{-1}}$. The initial double $\boldsymbol{q}$-vector helical state returns upon cooling below T_C. Scalebars 1 µm.

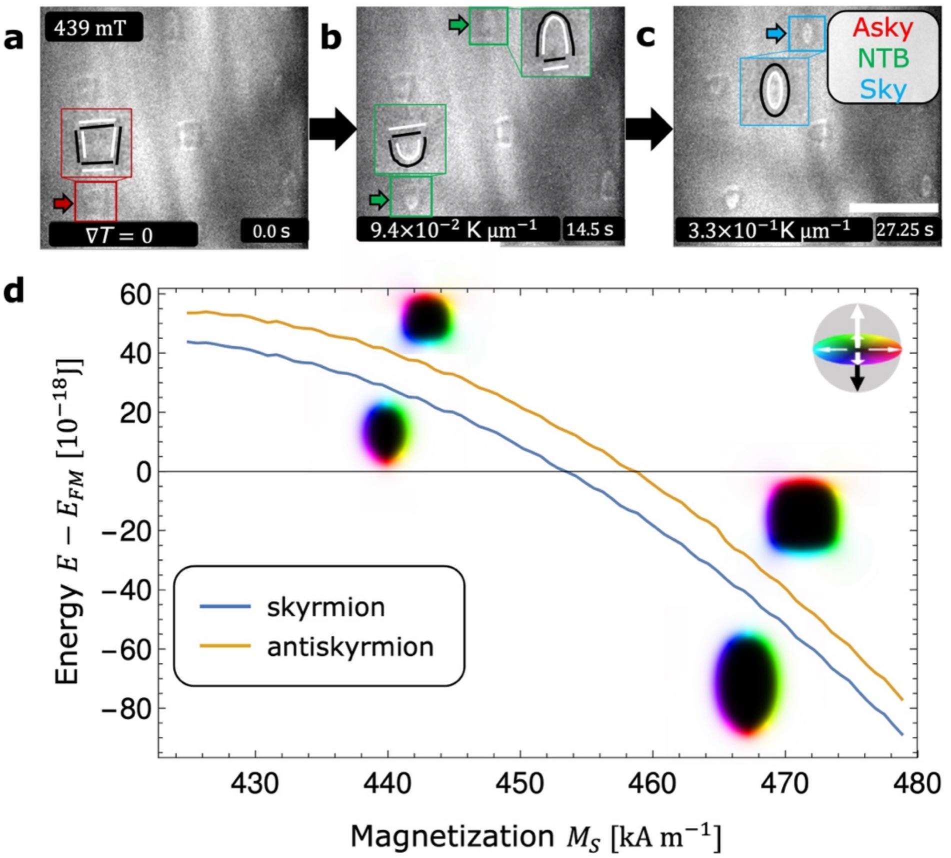

Figure S5: Topological spin texture transformation under an external magnetic field. **a**, LTEM micrographs of Askys and NTBs stabilized via an external magnetic field with magnitude $|B_z| = 439$ mT. **b-c**, Transformation of the initial magnetic spin textures shown in **a** into the spin textures shown in **b-c**, respectively. Selected Asky, NTB and Skys are indicated by red, green, and blue arrows, respectively. Insets show magnified images of selected spin textures with white and black lines drawn marking the bright and dark intensities in the LTEM micrographs. The heater current is increased linearly over a 30 s period and then held at a maximum for 30 s, with the frames in **a-c** taken while the heater current is increasing. The time stamps and approximate ∇T values are shown in the lower right and lower left of the panels, respectively. The maximum $\nabla T = 3.3 \times 10^{-1}$ K μm^{-1} corresponds to a maximum temperature $T = 302$ K in the field of view. The heater is located on the right-hand side of each image, while the cold bath is on the LHS. Scale bars are 0.5 μm. **d**, Energy of an isolated skyrmion (blue) and antiskyrmion (yellow) with respect to the polarized state as function of the magnetization (M_S), calculated via micromagnetic simulations at $|B_z| = 439$ mT. The micromagnetic interaction parameters are rescaled as functions of the magnetization, see Methods. The insets show the real-space texture of the elliptical skyrmion (lower) and square-shaped antiskyrmion (upper panels) at $M_S = 423.25$ kA m^{-1} and $M_S = 465.5$ kA m^{-1}, respectively and the colour wheel indicates the three-dimensional magnetization orientation.

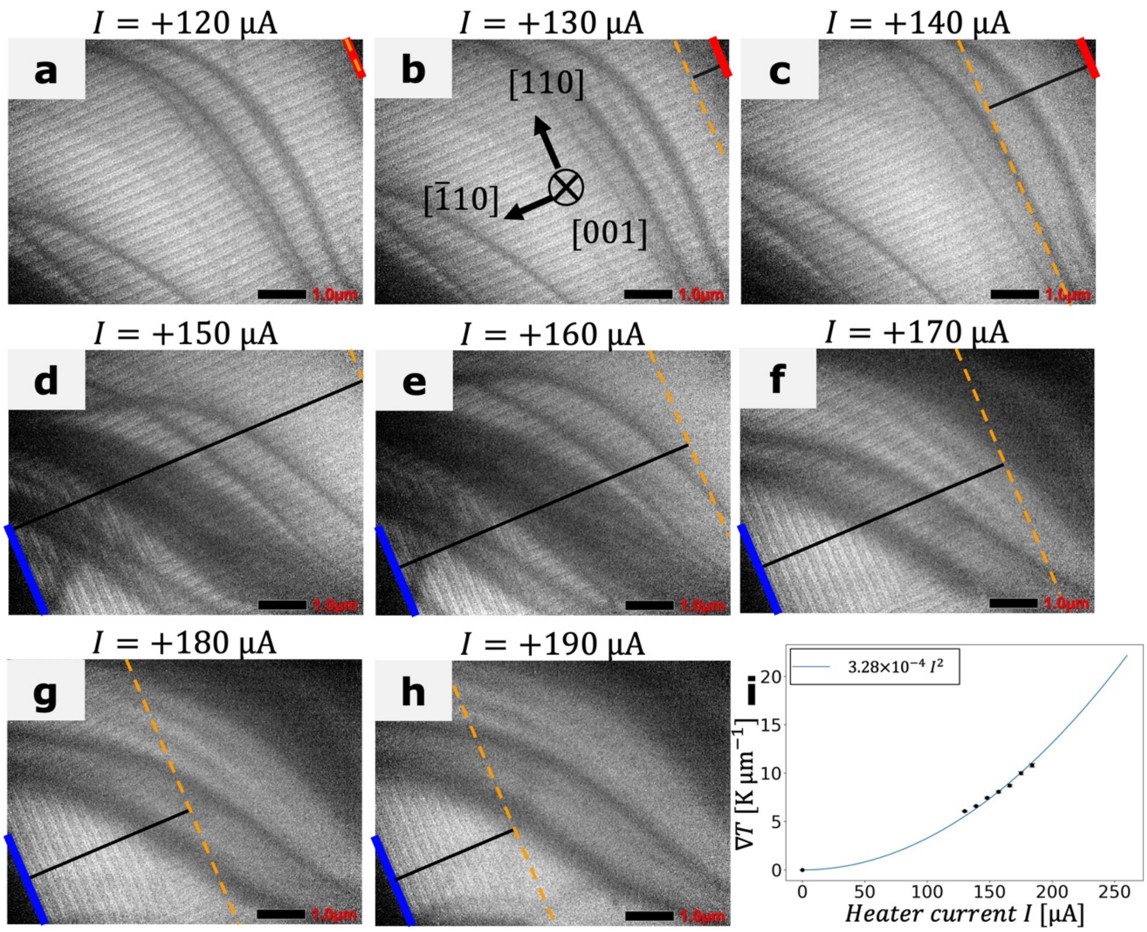

Figure S6. Estimation of ∇T via magnetic contrast measurement ($\mathcal{V}$) vs heater current I. **a-h**, Defocused LTEM micrograph of the helical domain at zero field for increasing values of I, labelled above each panel. The rectangular device has long axis parallel to the $[\bar{1}10]$ crystal axis (labelled in **b**) with the Pt heater wire located on the right-hand-side of the interface indicated by a solid red line in **a-c**. The solid blue line on the left-hand-side of **d-h** indicate the sample edge that is contacted to the cold bath held at room temperature. The dashed orange line in panels **b-h** indicate the line at which magnetic contrast is lost at $T \geq T_C = 362\ K$. **i**, $\nabla T\ [K\ \mu m^{-1}]$ vs $I\ [\mu A]$ estimated from **a-h**, with a quadratic fit shown by a solid blue line and error bars representing the standard deviation $\sigma_{\nabla T}$ assuming the uncertainty of each x-location of T_C measured for **a-i** is equal to a helical period $\sigma_{x_{T_C}} = 200\ nm$. The scale bars are 1 μm.

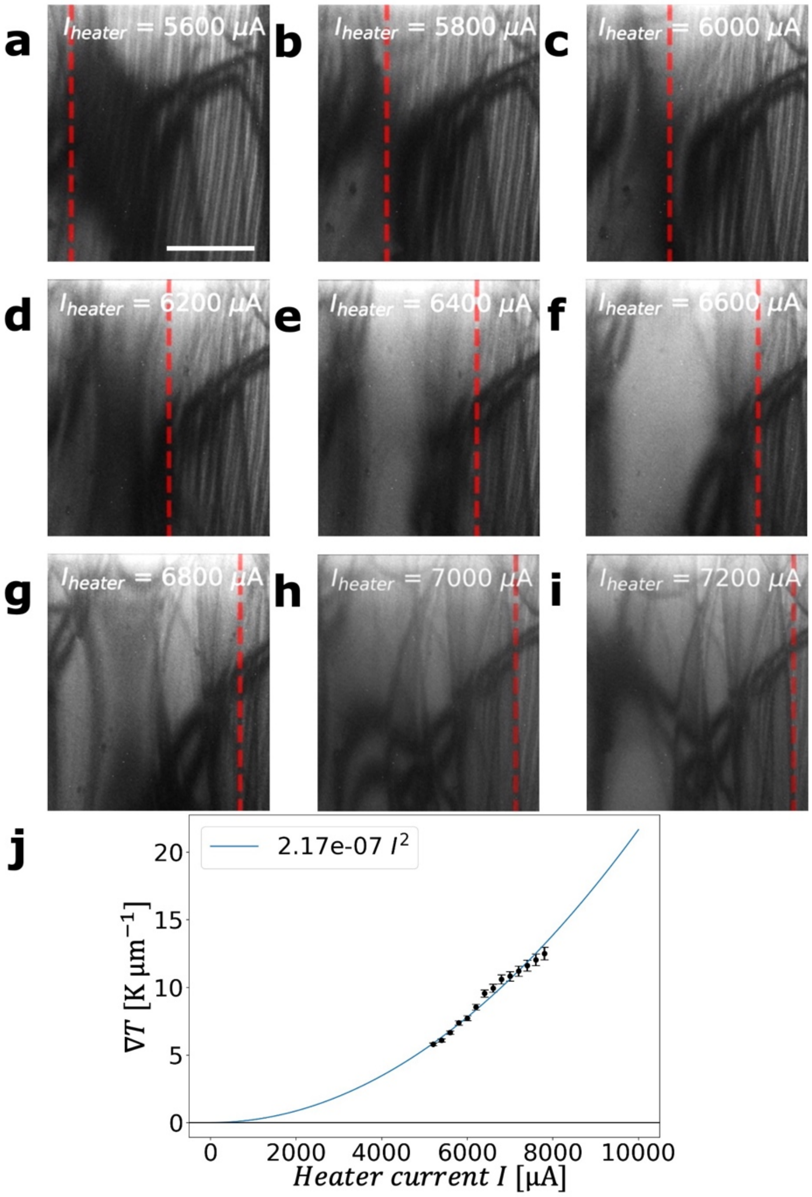

Figure S7. Estimation of ∇T via magnetic contrast measurement ($\mathcal{V}$) vs heater current I. a-i, Defocused LTEM micrograph of the helical domain at zero field for increasing values of I, labelled at the top of each panel. The rectangular device's long axis is parallel to the $[\bar{1}10]$ crystal axis (horizontal axis) with the Pt heater wire located on the left-hand-side of the field of view. The dashed red line in panels **a-i** indicate the line at which magnetic contrast is lost at $T \geq T_C = 362\ K$. **j**, $\nabla T\ [K\ \mu m^{-1}]$ vs $I\ [\mu A]$ estimated from **a-i**, with a quadratic fit shown by a solid blue line and error bars representing the standard deviation $\sigma_{\nabla T}$ assuming the uncertainty of each x-location of T_C measured for **a-i** is equal to a helical period $\sigma_{x_{T_C}} = 200\ nm$. Scale bar in **a** is 2 μm.
