# Supplementary material for: Heat current-driven topological spin texture transformations and helical q-vector switching
Source: Nat Commun. 2023 Nov 4;14:7094. doi: 10.1038/s41467-023-42846-7 (PMC10625536; doi:10.1038/s41467-023-42846-7)
Supplement: Supplementary file 2 — Description of Additional Supplementary Files [file 41467_2023_42846_MOESM2_ESM.pdf]

Title: Supplementary Movie 1:

Description: Temperature gradient driven transformation from skyrmion to antiskyrmion via a nontopological bubble at zero field. Live recording of a defocused LTEM micrograph showing a metastable skyrmion embedded in a helical background state under the application of constant $\boldsymbol{\nabla} T = 3.8\ \mathrm{K}\ \mu\mathrm{m}^{-1}$. The skyrmion transforms into an intermediate nontopological bubble before transforming again into an antiskyrmion. Field of view is $1.88\ \mu\mathrm{m}\ \times\ 1.06\ \mu\mathrm{m}$.

Title: Supplementary Movie 2:

Description: Uniform heating of metastable skyrmions at zero field. Left panel: real space LTEM micrographs showing the evolution of the spin texture population under the application of uniform heating, scalebar $1\ \mu\mathrm{m}$. Right panel: discrete Fourier transform of the LTEM micrographs cropped to highlight the relevant spin texture frequencies, scalebar $5\ \mu\mathrm{m}^{-1}$. The initial magnetic state contains metastable skyrmions, antiskyrmions and nontopological bubbles embedded in a magnetic helical background state at $T = 300\ \mathrm{K}$. After an initial increase to $T = 305\ \mathrm{K}$, the temperature is increased in $1\ \mathrm{K}$ steps up to $T = 361\ \mathrm{K}$. The temperature was held for $30 - 60$ s to allow the spin textures to stabilize before acquiring a real space image and increasing the temperature further.
